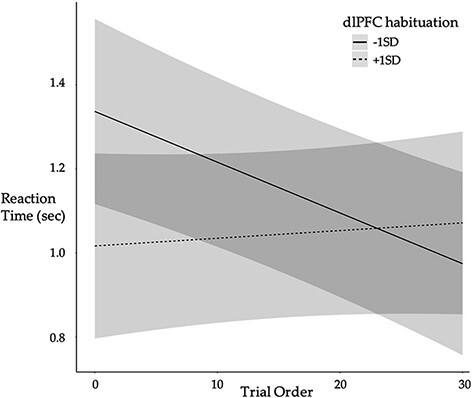# Corrigendum to: Dorsolateral prefrontal cortex response to negative tweets relates to executive functioning

**DOI:** 10.1093/scan/nsab039

**Published:** 2021-03-29

**Authors:** Sarah M Tashjian, Adriana Galván

**Affiliations:** Department of Psychology, University of California, Los Angeles, CA 90095, USA; Department of Psychology, University of California, Los Angeles, CA 90095, USA; Brain Research Institute, University of California, Los Angeles, CA 90095, USA

## Introduction

In the originally published version of this manuscript, Figure 7 inadvertently depicted vmPFC activation instead of depicting dlPFC habituation and the reaction time across the course of the Tweet Task.

All interpretations in the manuscript remain unchanged. Figure 7 has been updated as follows

Previous version

**Figure:**
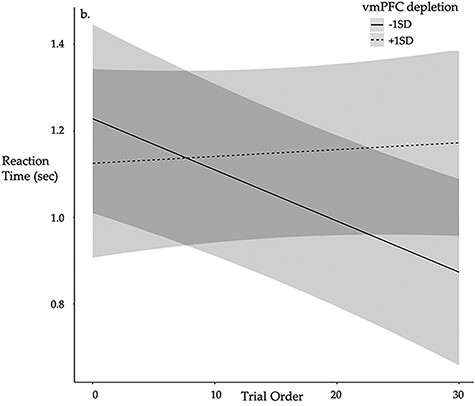


Corrected version

**Figure:**